# Contemporary management of sarcoidosis

**DOI:** 10.3389/fmed.2026.1878683

**Published:** 2026-07-29

**Authors:** Ngu Wah Khine, Sophie Fletcher

**Affiliations:** 1 Department of Respiratory Medicine, University Hospital Southampton NHS Foundation Trust, Southampton, United Kingdom; 2 School of Clinical and Experimental Sciences, Faculty of Medicine, University of Southampton, Southampton, United Kingdom; 3 NIHR Southampton Clinical Research Facility, Southampton, United Kingdom

**Keywords:** sarcoidosis, corticosteroids, immunosuppression, management, steroid-sparing therapy, emerging therapies

## Abstract

Sarcoidosis is a heterogeneous multisystem granulomatous disease with a highly variable clinical course ranging from spontaneous resolution to progressive organ dysfunction and life-threatening complications. Treatment decisions are guided by symptom burden, organ involvement, risk of irreversible damage, and overall impact on patient health-related quality of life. Corticosteroids remain the cornerstone of therapy; however, concerns regarding long-term cumulative toxicity and limited effects on disease progression have driven increasing interest in steroid-sparing and targeted therapeutic approaches. Recent clinical trial data evaluating earlier use of methotrexate and evolving steroid-sparing strategies reflect a changing treatment paradigm in selected patients with sarcoidosis.This review provides an updated overview of contemporary sarcoidosis management, integrating current guideline recommendations, recent clinical trial data, steroid-sparing approaches, biologic therapies, antifibrotic treatment, and emerging targeted therapies. In addition, current challenges in disease monitoring, treatment selection, and future directions toward personalized medicine are discussed.

## Introduction

Sarcoidosis is a complex multisystem granulomatous disease of unknown etiology, characterized by the formation of non-necrotising granulomas in affected organs. Although pulmonary involvement is the predominant manifestation, sarcoidosis can involve almost any organ system, resulting in a highly variable clinical presentation and disease course. Approximately 50–70% of patients undergo spontaneous resolution, whereas 10–30% develop persistent or progressive disease, irreversible organ dysfunction, and life-threatening cardiac and neurological involvement, which are associated with significant morbidity and mortality (1–3).

While sarcoidosis affects individuals of all ages, it occurs most frequently in young and middle-aged adults. A bimodal age distribution has been described, with a second peak in older adults, particularly among women (4, 5). The incidence of sarcoidosis varies widely by race, ethnicity and geographic region (6, 7). Recent population-based data from England demonstrated an increase in incidence from 6.65 to 7.73 per 100,000 person-years between 2003 and 2023, accompanied by a corresponding rise in mortality (8).

Despite being first described in the late 19th century based on its cutaneous manifestations, sarcoidosis remains a complex disease with ongoing challenges in diagnosis and management (2, 5). Current guidelines recommend corticosteroids as first-line therapy for patients requiring treatment (9–11). However, increasing recognition of corticosteroid-related toxicity, prolonged treatment requirements, frequent relapse during tapering or withdrawal, and limited evidence regarding long-term disease modification have contributed to growing interest in steroid-sparing approaches and evolving treatment paradigms in sarcoidosis management (9, 10, 12). Recent clinical trial data evaluating methotrexate as an earlier treatment option in pulmonary sarcoidosis, together with growing evidence supporting biologic and targeted therapies, reflect evolving therapeutic strategies in sarcoidosis management (13, 14). Given this evolving therapeutic landscape and the increasing complexity of treatment decision-making, there remains a need for an updated review of current evidence to guide sarcoidosis management. This review examines contemporary treatment approaches, and emerging targeted therapies, while also addressing current challenges in disease monitoring and future directions.

Literature search: We searched PubMed (MEDLINE) and Google Scholar for relevant English-language publications up to June 2026. No publication date restriction was applied, allowing inclusion of both landmark and contemporary studies relevant to the diagnosis and management of sarcoidosis. Conference abstracts were excluded.

## Disease overview

Although the exact etiology of sarcoidosis is unclear, it is thought to result from an abnormal immune response to environmental or infectious triggers in susceptible individuals (15–17). Genetic susceptibility plays an important role, with associations identified in both human leucocyte antigen (HLA) and non-HLA alleles (18, 19). In particular, HLA-DRB1 variants have been associated with disease susceptibility, clinical phenotype, and disease course (20)

Exposure to potential triggers leads to activation of innate immune cells, including macrophages and dendritic cells, followed by activation of CD4+ T lymphocytes and a predominantly T helper type 1 (Th1)-mediated immune response. This process is characterized by the production of pro-inflammatory cytokines, including interferon-γ and tumor necrosis factor-α (TNF-α), which contribute to granuloma formation and maintenance (17). Granuloma formation represents the hallmark histopathological feature of sarcoidosis and supports the diagnosis on tissue biopsy (15). Persistent granulomatous inflammation may result in chronic inflammation and irreversible fibrosis, and the mechanisms underlying this process remain uncertain, likely involving dysregulation of Th1, Th17, profibrotic Th2, and regulatory immune pathways (17). Increasing understanding of the immunological pathways involved in sarcoidosis has contributed to growing interest in targeted therapeutic approaches, including biologic therapies and emerging molecular-targeted treatments.

## Clinical presentation

Sarcoidosis has a broad spectrum of clinical manifestations. Approximately 30–50% of patients are asymptomatic at diagnosis (1), whereas others present with variable systemic and organ-specific manifestations depending on disease severity, extent of organ involvement, and patient-related factors such as age, ethnicity, and comorbidities (21, 22).

Acute sarcoidosis may present as Löfgren’s syndrome, characterized by erythema nodosum, bilateral hilar lymphadenopathy, arthritis, and occasionally fever or uveitis, and is generally associated with a favorable prognosis (23). Systemic symptoms such as fatigue, fever, night sweats, and weight loss are also frequently reported. Fatigue is one of the most frequently reported systemic symptoms of sarcoidosis and contributes to symptom burden and impaired quality of life (24).

Hypercalcaemia and hypercalciuria are recognized metabolic complications of sarcoidosis and may increase the risk of nephrolithiasis and renal dysfunction (25). Dysregulated vitamin D metabolism by activated macrophages within granulomas contributes to increased intestinal calcium absorption. Patients may present with fatigue, polyuria, nephrolithiasis, or renal dysfunction (26). The presence of hypercalcaemia has important implications for management, particularly when considering calcium and vitamin D supplementation.

Pulmonary involvement, including mediastinal and hilar lymphadenopathy, is the most common feature of sarcoidosis, occurring in approximately 90% of patients (22, 26). Clinical presentation ranges from incidental radiographic findings in asymptomatic individuals to progressive respiratory failure in advanced disease. Symptoms are usually non-specific, with dyspnoea and cough being the most common manifestations (27). Individuals of Black ethnicity are more likely to develop severe pulmonary disease and chronic extrapulmonary involvement (21, 28, 29). Data from a large United States cohort demonstrate that Black patients present with significantly more advanced radiographic disease, greater extrapulmonary burden (mean: 2.48 vs. 2.06 organs), and are more likely to require anti-sarcoidosis therapy (74.5% vs. 55.1%) compared with White counterparts (21). Longitudinal studies further highlight these disparities, revealing greater pulmonary function decline and an approximately 12-fold higher age-adjusted mortality among Black populations (29). Similar ethnic variations have also been reported in Hispanic and Asian populations; however, the available evidence remains less extensive, and phenotypic expression varies considerably across geographic regions (29). These ethnic variations may reflect a complex interplay between genetic susceptibility and socioeconomic factors, including reduced access to specialist care, which can contribute to delays in diagnosis and treatment escalation (28, 29).

Chest radiographic findings are classically classified according to the Scadding staging system based on the presence of lymphadenopathy and parenchymal involvement, and this staging system provides prognostic information (Table 1). However, chest radiography has recognized limitations and does not reliably reflect disease activity, symptom burden, or long-term prognosis (30, 31).

**TABLE 1:** Chest radiographic (Scadding) staging of pulmonary sarcoidosis and associated rates of spontaneous resolution.

Radiographic stage	Radiographic features	Frequency (%)	Spontaneous resolution (%)
0	Normal chest radiograph	5–15%	–
I	Bilateral hilar lymphadenopathy only	25–65%	60–90%
II	Bilateral hilar lymphadenopathy with Pulmonary infiltrates	20–40%	40–70%
III	Pulmonary infiltrates without bilateral hilar lymphadenopathy	10–15%	10–30%
IV	Pulmonary fibrosis	5%	0

Patients with persistent pulmonary inflammation, advanced radiographic disease, or chronic multisystem involvement are at greater risk of developing progressive fibrotic sarcoidosis. Pulmonary fibrosis develops in 10-20% of pulmonary sarcoidosis cases and is associated with increased morbidity and mortality (32, 33). Patients with progressive fibrotic disease require closer longitudinal monitoring and multidisciplinary management because of irreversible lung impairment and progressive respiratory dysfunction (34). Sarcoidosis-associated pulmonary hypertension (SAPH) is an uncommon but important complication of sarcoidosis, occurring in approximately 3% of patients overall (35, 36). In patients with stage IV fibrotic pulmonary sarcoidosis, pulmonary hypertension develops in approximately 30% of cases (37). SAPH is associated with increased morbidity and mortality and should be suspected in patients with exertional dyspnoea that is disproportionate to the extent of parenchymal lung disease, or markedly impaired gas transfer (37, 38).

## Extrapulmonary sarcoidosis

**FIGURE 1:**
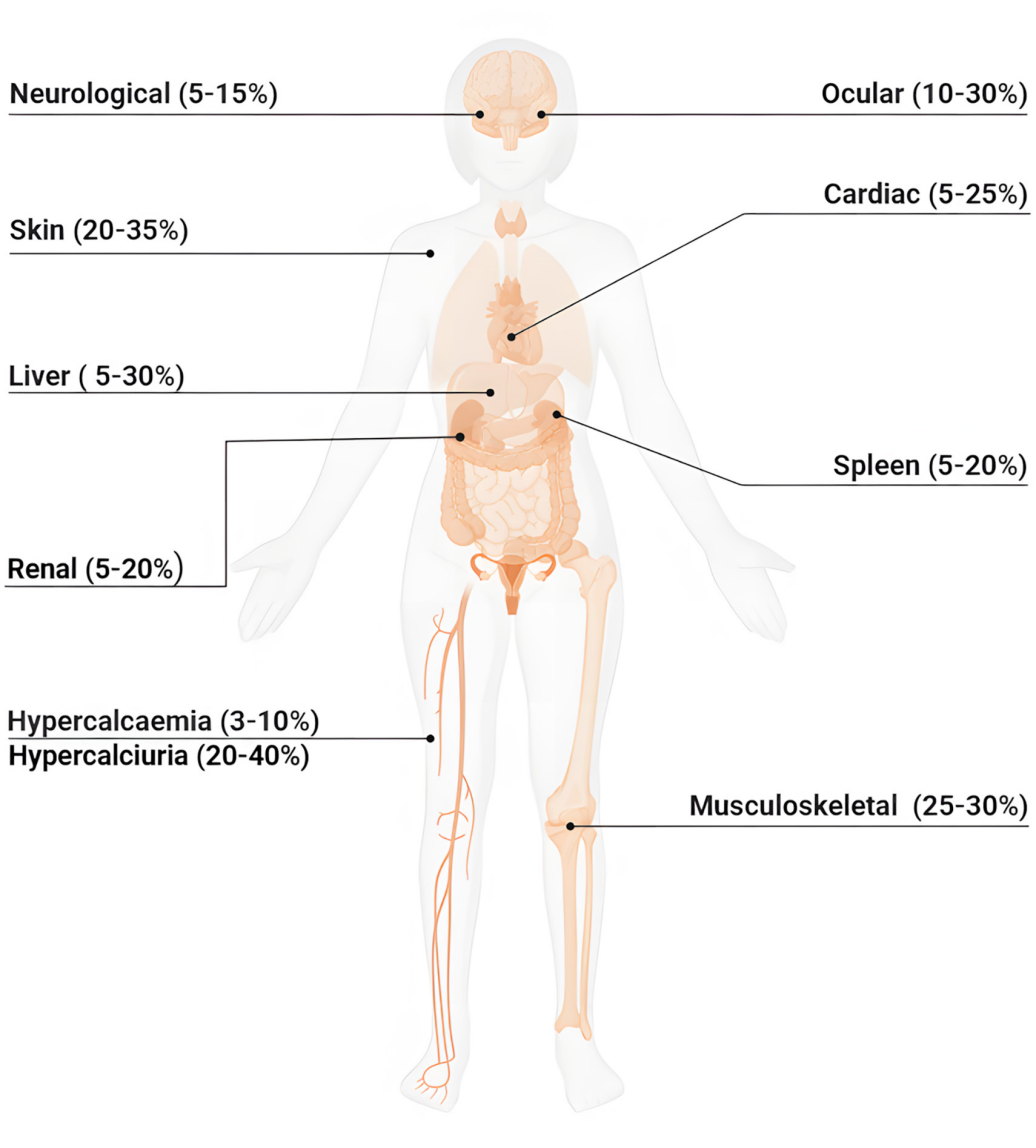
Estimated prevalence of common extrapulmonary manifestations of sarcoidosis. Data are synthesized from published literature. Reported prevalence rates vary according to ethnicity, geographic region, and screening methodology. Note that hypercalcaemia and hypercalciuria represent calcium metabolism disturbances associated with sarcoidosis rather than direct organ involvement (25, 39–46). Created in BioRender. Khine, N. (2026) https://BioRender.com/n9lc0bv (Agreement number: BB29ZGNM8N).

Approximately half of patients develop extrapulmonary disease, though reported frequencies vary between populations and study cohorts. Extrapulmonary involvement may affect multiple organ systems with skin, eyes, and lymph nodes most commonly affected. Cardiac and neurological sarcoidosis occur less frequently but warrant particular attention because of their association with adverse outcomes (26). The reported prevalence of major extrapulmonary manifestations is summarized in Figure 1.

Cutaneous manifestations include erythema nodosum, which is often associated with acute, self-limiting disease, and lupus pernio, which is more frequently associated with chronic, progressive disease and poorer prognosis (47). Ocular involvement most commonly presents as uveitis (48). Early recognition is important, as untreated ocular sarcoidosis may result in visual impairment and permanent vision loss (48).

Although hepatic and splenic involvement are relatively common manifestations of sarcoidosis, both are frequently asymptomatic and may therefore be under-recognized in routine clinical practice (40, 49, 50). They are often detected incidentally on imaging or routine clinical assessment (45). Hepatic sarcoidosis may present with hepatomegaly, cholestatic liver enzyme abnormalities, portal hypertension, or, rarely, cirrhosis (49, 50). Features of clinically apparent splenic sarcoidosis include splenomegaly, abdominal discomfort, and cytopenias, particularly in patients with multisystem diseases (40).

Cardiac involvement in sarcoidosis may result in conduction abnormalities, ventricular and supraventricular arrhythmias, heart failure, or sudden cardiac death and often requires aggressive immunosuppressive treatment and multidisciplinary management (43, 51). Although clinically apparent cardiac sarcoidosis occurs in approximately 10% of patients, advanced imaging techniques have identified subclinical cardiac involvement in up to 25% of patients (43). Overall, cardiac sarcoidosis remains a major contributor to sarcoidosis-related mortality, highlighting the importance of early recognition and timely intervention (43, 51)

Neurosarcoidosis can involve any part of the central or peripheral nervous system and presents with a broad spectrum of neurological manifestations. Cranial neuropathies are most common (55%), particularly involving the facial (24%) and optic nerves (21%) (52). Other manifestations include chronic meningitis, seizures, myelopathy, peripheral neuropathy, and hypothalamic–pituitary dysfunction (44). Its heterogeneous presentation and potential for long-term neurological impairment contribute substantially to disease-related morbidity (44).

## Clinical course

The clinical course of sarcoidosis is highly variable and often difficult to predict. Although many patients experience spontaneous remission, approximately one-third develop chronic or progressive disease requiring long-term treatment and monitoring (16). Persistent inflammation may lead to irreversible organ dysfunction, fibrotic lung disease, and long-term morbidity. Relapsing disease is common, particularly during corticosteroid tapering or treatment withdrawal, and some patients demonstrate ongoing subclinical inflammatory activity despite apparent clinical stability. Although the overall prognosis is favorable in most patients, mortality is increased in those with advanced pulmonary fibrosis, cardiac sarcoidosis or neurosarcoidosis (1, 3). These variations in disease behavior continue to present major challenges in prognostication and long-term management.

## Diagnosis

The diagnosis of sarcoidosis is based on compatible clinical and radiological features, supported by histologic evidence of non-necrotising granulomas and the exclusion of differential diagnoses (11). Clinical assessment should include symptom burden, organ involvement, and possible alternative diagnoses. Baseline investigations typically include routine blood tests, calcium studies, pulmonary function testing, and electrocardiography (9, 10). Laboratory investigations have more of a monitoring role than diagnostic and may include serum angiotensin-converting enzyme (ACE) and soluble interleukin-2 receptor (sIL-2R) levels. However, no biomarker has sufficient sensitivity or specificity to establish the diagnosis independently (53).

Imaging plays a central role in the diagnosis and assessment of sarcoidosis. While chest radiography is often the initial investigation, its sensitivity for detecting mediastinal lymphadenopathy and subtle parenchymal abnormalities is limited. High-resolution computed tomography (HRCT) is more sensitive than chest radiography for detecting mediastinal and hilar lymphadenopathy and characterizing parenchymal abnormalities (54, 55). Typical HRCT features include bilateral hilar and mediastinal lymphadenopathy with perilymphatic nodules, often with an upper lobe predominance (55). Fluorodeoxyglucose positron emission tomography-computed tomography (FDG PET-CT) may provide additional information on disease activity and the extent of organ involvement. A recent meta-analysis reported a pooled sensitivity of approximately 97% and specificity of 87% for pulmonary sarcoidosis (56). It can help guide treatment in complicated cases or when decisions about treatment changes are needed (56). The ERS guideline recommends FDG PET-CT as an adjunct for assessing disease activity in persistent cases, detection and monitoring cardiac disease, and guiding biopsy (9). Magnetic resonance imaging (MRI) has a limited role in pulmonary sarcoidosis, but is valuable for assessing cardiac and neurological involvement (54). Compared with FDG PET-CT, cardiac MRI has a higher reported sensitivity (approximately 95% versus 84%) and specificity (approximately 85% versus 82%) in cardiac sarcoidosis (57). The British Thoracic Society (BTS) clinical statement recommends cardiac MRI as the preferred investigation in patients with suspected cardiac sarcoidosis, with FDG PET-CT used as an adjunct (10).

Following clinical assessment and imaging, the need for histological confirmation should be considered on an individual basis. Histological confirmation is recommended in most patients, particularly when diagnostic uncertainty exists or alternative diagnoses need to be excluded (9–11). However, a biopsy may not be required in selected patients with characteristic presentations such as Löfgren syndrome or when a confident diagnosis can be reached through multidisciplinary assessment (9–11, 58). When tissue biopsy is required, the preferred biopsy site is the safest and most accessible involved organ. Endobronchial ultrasound-guided transbronchial needle aspiration (EBUS-TBNA) is generally preferred for mediastinal and hilar lymph node sampling because of its high diagnostic yield, reported at approximately 74.5%, and favorable safety profile (11, 59). In patients with predominantly parenchymal disease, transbronchial lung biopsy may be considered (9–11). Bronchoalveolar lavage (BAL) lymphocytosis may provide supportive evidence for diagnosis sarcoidosis, but it is non-specific (60).

## Management

Management of sarcoidosis should be individualized. The aim of treatment is to improve quality of life, prevent irreversible organ damage and minimize treatment-related toxicity. Not all patients require systemic therapy; careful observation is recommended for asymptomatic, mild or self-limiting disease, whereas treatment is indicated for significant symptom burden, progressive pulmonary disease, impaired organ function, or potentially life-threatening involvement such as cardiac, neurological, or severe ocular disease (9, 61, 62). Timely recognition of patients who require treatment is important to prevent disease progression and organ dysfunction. Treatment decisions should therefore balance the expected benefits of immunosuppression against the risks of therapy and be made through shared decision-making with the patient (63). Given its multisystem nature, complex sarcoidosis is best managed through a multidisciplinary team (MDT) approach to optimize treatment, monitoring, and quality of life (63)

Löfgren syndrome is a distinct acute clinical phenotype of sarcoidosis that is typically self-limiting (64). Consistent with current guideline recommendations, the majority of patients can be managed with observation and symptomatic treatment alone, including non-steroidal anti-inflammatory drugs for arthralgia and constitutional symptoms (9, 10). However, patients with persistent, severe or refractory symptoms may require treatment escalation to systemic corticosteroids or other immunomodulatory therapies (63). Despite its overall favorable prognosis, approximately 10–20% of patients may develop a more chronic disease course, although estimates vary between studies (64, 65).

## Pulmonary sarcoidosis

In pulmonary sarcoidosis, immunosuppressive therapy is most beneficial in patients with active inflammatory disease, significant functional impairment, or evidence of progression (9, 10), whereas its role is limited in established fibrotic disease (34, 66). Table 2 summarizes the principal immunosuppressive therapies used in sarcoidosis, which are discussed in greater detail in subsequent sections. Antifibrotic therapy may have a role in patients with progressive fibrotic pulmonary sarcoidosis. Antifibrotic agents such as nintedanib act through the inhibition of multiple tyrosine kinases involved in fibrogenic pathways, thereby slowing the progression of lung fibrosis (34, 66).

**TABLE 2:** Overview of pharmacological therapies in the management of sarcoidosis.

Drug	Dosage	Important adverse effects	Monitoring	Recommendation & references
Glucocorticoids	Prednisolone 20–40/day, Taper to ≤10/day	• Mood changes • Insomnia • Hypertension • Diabetes • Weight gain • Osteoporosis/fracture • Infections	Blood pressure, glucose, bone density, ophthalmology assessment where indicated	First line therapy for symptomatic, progressive, or major organ-threatening disease (9, 10, 12)
Methotrexate	10–25 mg once weekly with folic acid	• Nausea • Fatigue, • Infections • Cytopenias • Hepatotoxicity	FBC, LFT, renal function at baseline; every 2-4 weeks during initiation/escalation, then every 2-3 months	Preferred second-line steroid-sparing agent; The PREDMETH trial supports consideration as an alternative first-line therapy in selected pulmonary sarcoidosis (9, 10, 13, 72)
Mycophenolate mofetil	500–1500 mg twice daily	• Diarrhea • Nausea • Cytopenias • Infections	FBC, LFT and renal function	Alternative second-line steroid-sparing agent, often considered in multisystem disease (9, 10)
Azathioprine	1.5–2.5 mg/kg/day	• GI upset • Cytopenias • Infections • Hepatotoxicity	FBC, LFT, TPMT test before initiation	Alternative second-line steroid-sparing agent when methotrexate not tolerated (9, 10)
Leflunomide	10–20 mg/day	• GI upset • Cytopenias • Infections • Hepatotoxicity	FBC, LFT and renal function test	Alternative second-line steroid-sparing agent when methotrexate not tolerated (Limited evidence) (9, 10)
Hydroxy chloroquine	200–400 mg/day	• Retinopathy • GI upset • Skin pigmentation	Baseline ophthalmological assessment and annual retinal screening after 5 years of therapy (earlier in high-risk patients)	Useful in cutaneous disease, musculoskeletal symptoms and hypercalcaemia; limited role in significant pulmonary disease (9, 10, 73)
TNF-α inhibitor Infliximab Adalimumab	Infliximab 3–5 mg/kg IV at weeks 0, 2, and 6, then every 4–8 weeks	• Infections • Antibody formation • Infusion reactions • Worsening of heart failure and demyelinating disease	Screen for latent tuberculosis and hepatitis B/C before treatment. Monitor infusion reactions and infections	Preferred biologic therapy for refractory or organ-threatening sarcoidosis after failure of conventional immunosuppression (9, 10, 74)
	Adalimumab 40 mg subcutaneously every 1–2 weeks			
Rituximab	1,000 mg IV on days 1 and 15; retreatment individualised according to clinical response	• Infection • Hypogammaglobulinaemia • Infusion reaction	Screen for latent tuberculosis, hepatitis B/C before treatment. Monitor infusion reactions and infections	Evidence limited (9)

FBC, full blood count; LFT, liver function tests; TB, tuberculosis; TNF-α, tumor necrosis factor-alpha; IV, intravenous, GI, gastrointestinal; TPMT; Thiopurine methyltransferase. Drug mechanisms of action, adverse effect profiles, dosing, and monitoring recommendations are adapted from Clinical pharmacology in sarcoidosis: How to use and monitor sarcoidosis medications (75) and Immunosuppressive therapies in pulmonary sarcoidosis (76). Clinical indications and management recommendations are supported by the current guidelines and drug-specific references cited in the final column.

Evidence from the INBUILD trial demonstrated that nintedanib significantly reduced the rate of decline in forced vital capacity (FVC) in patients with progressive fibrosing interstitial lung diseases compared with placebo (67). However, only a small proportion of patients with sarcoidosis were included in the study, limiting disease-specific conclusions. Therefore, the role of antifibrotic therapy in sarcoidosis remains uncertain and has yet to be established in dedicated sarcoidosis-specific clinical trials (34, 67). In advanced fibrotic pulmonary sarcoidosis, management is largely supportive and includes pulmonary rehabilitation, oxygen therapy where indicated, and treatment of complications such as SAPH. According to the World Association of Sarcoidosis and Other Granulomatous Disorders (WASOG) statement, patients with SAPH should be managed by a multidisciplinary team with expertise in both sarcoidosis and pulmonary hypertension. Off-label pulmonary arterial hypertension-targeted therapy may be considered for selected symptomatic patients on a case-by-case basis (38). Lung transplantation may be considered in carefully selected patients with end-stage pulmonary sarcoidosis, particularly those with progressive respiratory failure, advanced fibrotic lung disease, or SAPH (34, 68, 69).

## Extrapulmonary sarcoidosis

Cardiac and neurological sarcoidosis generally require prompt systemic therapy and coordinated multidisciplinary management because of the risk of irreversible organ damage and life-threatening complications. Given the prolonged treatment often required, early introduction of steroid-sparing immunosuppressive therapy is frequently considered to facilitate corticosteroid tapering and minimize treatment-related toxicity (9, 43, 52). Selected patients with refractory disease may require escalation to biologic (9)

Ocular sarcoidosis may be managed with topical therapy when anterior disease is mild, but systemic treatment is required for severe, posterior, refractory, or sight-threatening disease. For cutaneous sarcoidosis, topical or intralesional corticosteroids are often sufficient for limited disease, whereas extensive, progressive, or significant skin disease may require systemic immunosuppressive therapy. Involvement of organs such as the liver or peripheral lymph nodes may not require treatment unless symptomatic, progressive, or associated with organ dysfunction (9).

Management of hypercalcaemia is guided by its severity and symptom burden. Alternative causes of hypercalcaemia, including malignancy and primary hyperparathyroidism, should first be excluded. In symptomatic or moderate-to-severe cases, systemic corticosteroids are the mainstay of treatment, with additional supportive measures such as intravenous fluids and, if required, bisphosphonates (25). In patients with recurrent or treatment-resistant hypercalcaemia, steroid-sparing agents such as hydroxychloroquine and methotrexate have been used, although supporting evidence remains limited (70). Regular monitoring of serum calcium levels and renal function is recommended. The use of calcium and vitamin D supplementation in patients receiving long-term corticosteroid therapy should be considered cautiously due to the increased risk of hypercalcaemia (71).

## Corticosteroids in sarcoidosis: clinical role and evidence base

Glucocorticoid therapy remains the cornerstone of treatment for sarcoidosis and is recommended as first-line therapy in current clinical practice guidelines (9–11). Their anti-inflammatory and immunosuppressive properties suppress granulomatous inflammation, thereby improving symptoms, organ function, and radiographic abnormalities in patients with active disease (75). However, their use requires balancing potential clinical benefits against the risks associated with prolonged treatment. Despite their widespread use, the evidence supporting corticosteroid therapy is based largely on improvements in short-term clinical and radiological outcomes, with less certainty regarding long-term disease modification and prevention of progression (77, 78).

Several studies have reported the short-term benefit of corticosteroids in patients with pulmonary sarcoidosis. A Cochrane systematic review of 12 randomised controlled trials (RCTs) found that oral corticosteroid therapy (4–40 mg/day) was associated with improvements in chest radiographic findings, global chest X-ray scores, and symptoms over 3–24 months. These benefits were modest, with limited evidence for sustained improvements in lung function and effects on long-term disease progression (77). Similarly, an earlier review reported improvements in radiographic outcomes over 6–24 months, with only small improvements in lung function in patients with stage II and III sarcoidosis (78). Collectively, evidence from RCTs and systematic reviews supports the short-term efficacy of corticosteroids in pulmonary sarcoidosis, although evidence for long-term disease modification and prevention of progression remains limited (77, 78).

In contrast, the evidence supporting inhaled corticosteroids in sarcoidosis is limited. A recent 2024 review found limited evidence supporting their use, with modest benefit reported mainly for budesonide as maintenance therapy following induction with systemic corticosteroids or in patients with relapse; however, routine use of inhaled corticosteroids in sarcoidosis is not currently supported by the available evidence (79). Although one prospective randomized study reported improved lung function at 5-year follow-up following early corticosteroid treatment and maintenance with inhaled budesonide, the study predominantly included patients with early-stage disease and preserved lung function, in whom spontaneous improvement is common, limiting the ability to attribute these benefits solely to inhaled budesonide (3, 5, 80).

## Corticosteroids dosing and treatment strategies

Current ERS guidance recommends an initial prednisolone dose of 20 mg/day, gradually tapered to the lowest effective maintenance dose, usually 5–10 mg/day (9). Several studies have questioned whether higher doses provide additional benefit. In the SARCORT randomized controlled trial, no significant difference was observed between 40 mg/day and 20 mg/day prednisolone in patients with sarcoidosis in terms of treatment failure, time to relapse, health-related quality of life, or lung function (81). Similarly, an observational study found no association between prednisone dose and changes in forced vital capacity in patients with pulmonary sarcoidosis initiating corticosteroid therapy, although higher cumulative doses were associated with significant weight gain. In addition, lower cumulative steroid exposure was largely achieved through earlier tapering to doses of ≤ 10 mg/day within 3.5 months of treatment initiation (82). These findings support the use of the lowest effective dose and early tapering strategies to minimize cumulative toxicity while maintaining disease control (81, 82).

## Toxicity and challenges of long-term corticosteroid therapy

Complications of corticosteroids include gastritis, weight gain, hypertension, diabetes mellitus, fluid retention, mood changes, increased risk of infection, and more serious long-term risks such as osteoporosis, Cushing’s syndrome, and adrenal insufficiency when glucocorticoids are tapered or withdrawn (81). The risk of adverse effects is influenced by corticosteroid dose, treatment duration, cumulative exposure, and patient factors such as comorbidities (83). Large cohort studies and meta-analyses have demonstrated an increased risk of fracture with systemic corticosteroid doses of ≥5 mg/day, with excess risk becoming evident within the first 3–6 months of treatment (84, 85).

In clinical practice, oral glucocorticoids can be difficult to discontinue once initiated. This likely reflects that patients requiring systemic therapy often have more severe or chronic disease, including major organ involvement, and are therefore more likely to require prolonged treatment (86). Patients who require treatment early in the disease course, within 6 months of diagnosis, are more likely to remain on therapy at follow-up, including beyond 2 years (87). In addition, relapse following corticosteroid-induced remission has been reported in up to 74% of patients, whereas relapse is less common in those with spontaneous remission (8%) (88). This highlights the importance of careful patient selection when considering the potential benefits against the risks of prolonged therapy and cumulative toxicity.

## Monitoring during corticosteroid therapy

In view of the well-recognized adverse effects associated with glucocorticoid therapy, baseline evaluation is advisable in patients expected to require longer-term treatment. Current guidance from the National Osteoporosis Guideline Group (NOGG) recommends early fracture risk assessment using FRAX in individuals receiving oral glucocorticoids for 3 months or more, and those identified as having intermediate or high fracture risk should have bone mineral density measurement by dual-energy X-ray absorptiometry (DEXA). In addition, patients receiving prednisolone-equivalent doses of ≥7.5 mg/day over a prolonged period may warrant bone-protective therapy (89). Gastrointestinal protection may be considered in patients at increased risk of peptic ulcer disease, particularly in those receiving prolonged corticosteroid therapy or concomitant NSAIDs (75). Prolonged corticosteroid therapy, particularly with additional immunosuppressive agents, is also associated with an increased risk of opportunistic infections, including *Pneumocystis jirovecii* pneumonia (PCP), in non-HIV immunocompromised patients. Evidence from a Cochrane systematic review demonstrates that trimethoprim-sulfamethoxazole prophylaxis significantly reduces the incidence of PCP and PCP-related mortality, although these data are largely derived from hematological malignancy and transplant populations rather than sarcoidosis-specific cohorts (90). Evidence supporting routine PCP prophylaxis specifically in sarcoidosis remains limited. Nevertheless, prophylaxis may be considered in selected patients receiving prolonged or high-dose corticosteroids, particularly at doses of ≥20 mg/day of prednisone for ≥4 weeks, especially when combined with additional immunosuppressive therapy (91). In clinical practice, decisions are therefore individualized based on the overall immunosuppressive burden.

## Steroid-sparing immunosuppressive therapy

Given the toxicity associated with prolonged corticosteroid therapy and the high rates of relapse following dose reduction or withdrawal, steroid-sparing immunosuppressive agents are increasingly used in patients requiring long-term treatment or in those with corticosteroid-related adverse effects (9, 75). Patients receiving immunosuppressive or biologic therapy are at increased risk of infection. Vaccination status should therefore be reviewed before commencing treatment, with age-appropriate immunisations administered before treatment whenever feasible to optimize vaccine responses (92). Inactivated vaccines can generally be administered safely during immunosuppressive therapy, whereas live attenuated vaccines should ideally be administered before treatment initiation and are generally avoided during periods of significant immunosuppression (92, 93).

In addition, patients should undergo baseline assessment, including a full blood count, renal and liver function tests, and screening for infections (75). Ongoing monitoring should be tailored to the specific agents used and is summarized in Table 1, with additional drug-specific recommendations discussed in the relevant sections below. Recommendations regarding the use of these agents during pregnancy are discussed in a later section.

## Methotrexate

Methotrexate (MTX) is the most extensively studied immunosuppressive agent in sarcoidosis, and is recommended by current guidelines as the preferred steroid-sparing therapy (9, 10). MTX exerts anti-inflammatory effects through several mechanisms, including the enhancement of adenosine signaling, resulting in suppression of T-cell-mediated inflammation (94).

The efficacy of MTX in sarcoidosis has been demonstrated in both clinical trials and real-world studies (95–97). A randomized controlled trial showed that methotrexate reduced cumulative corticosteroid exposure while maintaining disease control and without increasing adverse events, establishing its role as an effective corticosteroid-sparing agent (96). Notably, MTX has a delayed onset of action, and its therapeutic effects may not become apparent for several months after treatment initiation (72, 96). Real-world data have further supported the efficacy and tolerability of MTX in pulmonary sarcoidosis, demonstrating improvements in radiographic and physiological outcomes as well as beneficial effects on patient-reported outcomes, including health-related quality of life (97).

Until recently, evidence was largely limited to studies evaluating methotrexate as adjunctive therapy. The recent open-label PREDMETH trial addressed this evidence gap by comparing methotrexate with prednisolone as first-line therapy in patients with pulmonary sarcoidosis. It demonstrated that methotrexate was non-inferior to prednisolone as first-line therapy, with similar improvements in lung function at 24 weeks, although prednisolone produced a more rapid improvement in forced vital capacity during the early treatment phase, with effects observed as early as four weeks. Adverse events were similar between treatment groups; however, methotrexate was associated with a distinct toxicity profile, characterized predominantly by fatigue or malaise, gastrointestinal symptoms such as nausea and reduced appetite, and liver enzyme abnormalities (13). These findings should be interpreted with caution, given the open-label design, short follow-up duration, inclusion of only patients with treatment-requiring pulmonary sarcoidosis, and the absence of patient-reported outcome measures (13).

While current guidelines recommend a stepwise approach for treatment, with corticosteroids as the first line followed by steroid-sparing agents (9, 10), earlier introduction of immunosuppressive therapy may be considered in selected patients with severe disease, particularly those with cardiac sarcoidosis (9–11, 98). Whether the earlier introduction of MTX improves outcomes in cardiac sarcoidosis remains uncertain. This evidence gap is being addressed by the ongoing CHASM CS-RCT, which compares glucocorticoid monotherapy with combination therapy, and the results are still pending (99).

In clinical practice, the recommended initial dose of MTX is 10–15 mg once weekly, which may be titrated to 20–25 mg weekly based on clinical response and tolerability. Folic acid supplementation is recommended to reduce methotrexate-related adverse effects, particularly gastrointestinal, mucosal, and hematological toxicity (75). Clinicians should be aware of clinically important drug interactions. Concomitant use of trimethoprim or trimethoprim-sulfamethoxazole should be avoided due to increased risk of severe bone marrow suppression, while concomitant use of non-steroidal anti-inflammatory drugs (NSAIDs) may enhance methotrexate toxicity, particularly in patients with impaired renal function (75).

## Alternative immunosuppressive agents

Alternative immunosuppressive agents, including azathioprine, mycophenolate mofetil (MMF), and leflunomide, are used as steroid-sparing therapies in patients who are intolerant of methotrexate or in whom methotrexate is ineffective or contraindicated (76, 100, 101). However, compared with methotrexate, which has the most robust evidence base among conventional steroid-sparing therapies, the evidence supporting azathioprine, MMF and leflunomide remains more limited.

Azathioprine is a purine synthesis inhibitor that has historically been used as an alternative steroid-sparing agent in sarcoidosis patients requiring prolonged corticosteroid treatment or corticosteroid-related toxicity (75). An early prospective study demonstrated that azathioprine in combination with prednisolone was associated with improvements in symptoms, lung function and radiographic findings in patients with chronic sarcoidosis, supporting its role as a corticosteroid-sparing agent (101) A large comparative study reported similar effects of azathioprine and methotrexate on lung function and corticosteroid-sparing efficacy. Nevertheless, azathioprine was associated with higher rates of infection (34.6% vs. 18.1%) and treatment discontinuation, which may limit its use in some patients (102) Consequently, although azathioprine remains an effective alternative, methotrexate is generally preferred when tolerated because of its more favorable safety profile (75, 102)

Mycophenolate mofetil (MMF) is another second-line immunosuppressive therapy used in patients with sarcoidosis who are intolerant of methotrexate or have multisystem disease (75). Compared with methotrexate, evidence supporting MMF is more limited and derives predominantly from retrospective studies. Available data suggest that MMF may reduce corticosteroid requirements, is generally well tolerated, and may help stabilize disease; however, its effects on pulmonary function have been inconsistent across studies (100, 103, 104). Interpretation of these findings is limited by the retrospective study design, small sample sizes, heterogeneous patient populations, and the frequent use of MMF in patients who had previously failed or were intolerant of other immunosuppressive therapies (100, 103, 104). In neurosarcoidosis, MMF has demonstrated clinical benefit; however, comparative data suggest that methotrexate may be associated with longer relapse-free survival and superior long-term disease control (105). Nevertheless, MMF appears to be an alternative therapeutic option in selected patients, although further comparative studies are needed.

Leflunomide is an immunomodulatory agent that inhibits dihydroorotate dehydrogenase, thereby suppressing de novo pyrimidine synthesis and lymphocyte proliferation (106). Data supporting leflunomide in sarcoidosis are relatively sparse. Retrospective studies have reported improvements in both pulmonary and extrapulmonary disease manifestations, together with reductions in glucocorticoid requirements, although adverse events occurred in approximately one-third of patients and resulted in treatment discontinuation in around 17% of cases in one large cohort (107, 108). Leflunomide is generally considered an alternative immunosuppressive therapy for patients who are intolerant of methotrexate and is recognized in current guidelines as a potential treatment option despite the limited evidence base (9, 10)

Hydroxychloroquine is an antimalarial agent with immunomodulatory effects that is used in selected manifestations of sarcoidosis, particularly cutaneous disease, musculoskeletal manifestations and sarcoidosis-associated hypercalcaemia (9, 75, 109). Its role in pulmonary sarcoidosis is limited, and it is widely considered less effective than conventional immunosuppressive therapies (9, 75, 109). Long-term treatment requires regular ophthalmological monitoring because of the risk of retinal toxicity, and the current Royal College of Ophthalmologists guidance recommends annual retinal screening after 5 years, or earlier in patients with additional risk factors (73).

The use of cyclophosphamide in sarcoidosis is generally limited to refractory organ-threatening disease, particularly neurosarcoidosis (76). In neurosarcoidosis, observational studies have suggested an association with lower relapse rates (110). However, significant toxicities limit its use, and it is generally reserved for exceptional cases in specialist centers (76).

## Biologic therapy and sarcoidosis

Targeted biologic therapies may be used in refractory sarcoidosis, most commonly tumor necrosis factor-alpha (TNF-α) inhibitors such as infliximab and adalimumab (9, 10). They exert their effects by inhibiting TNF-α, a cytokine produced during macrophage activation that is responsible for granuloma formation (111). Among the available biologic agents, infliximab has the strongest evidence base and is the most extensively studied biologic therapy in sarcoidosis. Randomized controlled trials predominantly involving patients with pulmonary sarcoidosis have demonstrated improvements in lung function, supporting its use in refractory disease (112, 113). It has also demonstrated clinical benefit in refractory extrapulmonary sarcoidosis, including in a randomized trial of extrapulmonary disease and a prospective cohort of patients with FDG-PET-positive refractory sarcoidosis (114, 115). Available data on adalimumab in sarcoidosis are largely derived from smaller studies, which have reported benefit in selected patients, particularly those with cutaneous disease (116). However, evidence supporting its use in pulmonary sarcoidosis remains less robust than that for infliximab (117). Nonetheless, it may be considered in selected patients when infliximab is not tolerated or unsuitable (118).

Despite these encouraging findings, there are several limitations. Improvements in lung function observed in clinical trials have generally been modest (pooled mean change in % predicted FVC 4.79%) (118). Relatively few randomized controlled trials have evaluated biologic therapies in sarcoidosis, and many are small and at risk of bias. Interpretation is further influenced by substantial heterogeneity in study populations, organ involvement and outcome measures. Furthermore, measurement of forced vital capacity has frequently been used as a primary endpoint despite its limited ability to capture the multisystem disease activity of sarcoidosis and patient-reported burden (118).

Rituximab is a monoclonal antibody that targets CD20-positive B lymphocytes, resulting in transient B-cell depletion and a secondary reduction in granulomatous inflammation (14). It has been investigated as salvage therapy in refractory sarcoidosis (75). To date, prospective clinical evidence remains limited to a single-arm study in patients with refractory pulmonary sarcoidosis, which demonstrated variable clinical responses and no statistically significant improvement in forced vital capacity (119). Although data from small observational studies and case reports have been reported, high-quality evidence remains limited (118). Therefore, adequately powered randomized controlled trials are required before rituximab can be considered for routine clinical use.

Before initiating biologic therapy, patients should be screened for latent tuberculosis, hepatitis B, and hepatitis C because of the risk of infection reactivation during treatment, with ongoing monitoring for infections, infusion or injection-related reactions, and other treatment-related adverse effects throughout the treatment (75).

## Pregnancy and sarcoidosis

Most women with sarcoidosis have successful pregnancies, and disease activity often remains stable or improves during pregnancy, although relapse may occur in the postpartum period, and maternal sarcoidosis has been linked to adverse pregnancy outcomes (120). Immunosuppressive therapy may be required to control disease activity during pregnancy when clinically indicated; however, it is important to recognize that not all immunosuppressive agents are safe for use during pregnancy.

Corticosteroids are generally considered safe in pregnancy, with only a small increased risk of orofacial clefts, without clear evidence of a substantial increase in adverse birth outcomes (121). Other agents, including hydroxychloroquine, azathioprine and TNF-α inhibitors, are generally considered compatible when clinically indicated. In contrast, methotrexate, mycophenolate mofetil, leflunomide and cyclophosphamide are contraindicated in both pregnancy and lactation because of their potential fetal and neonatal toxicity (75, 120).

For women of childbearing age, it is essential for clinicians to explore future pregnancy intentions before initiating potentially teratogenic immunosuppressive therapies as discussed above. Appropriate counseling regarding contraception and pre-conception planning is essential, and recommended washout periods should be observed before conception (122). Therefore, multidisciplinary management is advocated to optimize both maternal and fetal outcomes.

## Fatigue in sarcoidosis

Fatigue is one of the most common and disabling symptoms of sarcoidosis, affecting up to 80% of patients and substantially impairing quality of life. Importantly, fatigue may persist despite disease control and does not necessarily correlate with objective markers of disease activity. Its etiology is multifactorial and may reflect ongoing inflammation, small-fiber neuropathy, sleep disorders, depression, anxiety, medication adverse effects, or comorbid conditions. Consequently, assessment should include evaluation for potentially reversible contributors before escalation of immunosuppressive therapy (123).

Management of sarcoidosis-associated fatigue remains challenging. Optimisation of disease control is important in patients with active inflammation; however, treatment of sarcoidosis alone is often insufficient to resolve fatigue (123). Non-pharmacological interventions, including structured exercise programmes, pulmonary rehabilitation, management of sleep disturbances and psychological interventions, are therefore central components of care (123, 124) Most recently, the TIRED randomized controlled trial demonstrated that online mindfulness-based cognitive therapy improved fatigue and quality-of-life measures in patients with sarcoidosis, supporting the role of psychological interventions in selected patients (124). Pharmacological approaches such as methylphenidate, dexmethylphenidate and armodafinil have demonstrated benefit in small studies, although the evidence base remains limited and routine use is not currently recommended (24, 123). Effective management requires a holistic, multidisciplinary approach. Current guidelines emphasize a systematic assessment for reversible causes of fatigue and recommend pulmonary rehabilitation or structured exercise-based interventions for patients with severe fatigue affecting quality of life (9).

## Multidisciplinary management and supportive care in sarcoidosis

Given the multisystem nature of sarcoidosis, management often requires a multidisciplinary team (MDT) approach. Depending on organ involvement, care may involve respiratory physicians, cardiologists, neurologists, ophthalmologists, dermatologists, rheumatologists, specialist nurses, and other allied health professionals. MDT input is particularly important in patients with complex multisystem disease, cardiac or neurological involvement, progressive fibrotic disease, or significant treatment-related toxicity (9, 10).

Supportive care is an integral component of sarcoidosis management and should address symptom burden, functional impairment, and quality of life (9). Pulmonary rehabilitation may improve exercise capacity, dyspnoea, fatigue, and health-related quality of life in selected patients (125). Regular follow-up and coordinated multidisciplinary care are important to optimize long-term outcomes (9, 10).

## Monitoring and follow-up

There is no universally accepted follow-up schedule because of the heterogeneous nature of sarcoidosis. The BTS Clinical Statement recommends that patients with stage I pulmonary sarcoidosis are typically followed for 2 years because of the high likelihood of spontaneous resolution, whereas those with persistent pulmonary disease or extrapulmonary involvement generally require longer-term follow-up (10).

Monitoring should be individualized according to organ involvement, risk of morbidity and mortality, symptom burden, quality of life, treatment requirements, and the likelihood of disease progression, with closer surveillance for patients with progressive pulmonary disease, major organ involvement, multisystem disease, or those receiving immunosuppressive therapy (9, 10). The intensity of monitoring may vary according to the treatment phase, with closer assessment often required during treatment initiation, dose escalation, and corticosteroid tapering than during stable maintenance therapy.

Follow-up should include assessment of symptom burden and functional status, pulmonary function testing, quality-of-life assessment, treatment-related adverse effects, and organ-directed investigations where appropriate to evaluate disease course, treatment response, and treatment-related toxicity (9, 10).

Current European Respiratory Society (ERS) guidance supports periodic reassessment of the need for ongoing treatment, with treatment withdrawal considered after 1–2 years of disease control because relapse following treatment discontinuation is common (9) Laboratory monitoring may be useful to assess organ involvement and treatment-related toxicity, including serum calcium, liver function tests, and renal function. While serum ACE may be used in clinical practice, its role in routine disease monitoring remains unclear (53)

## Discussion

**FIGURE 2:**
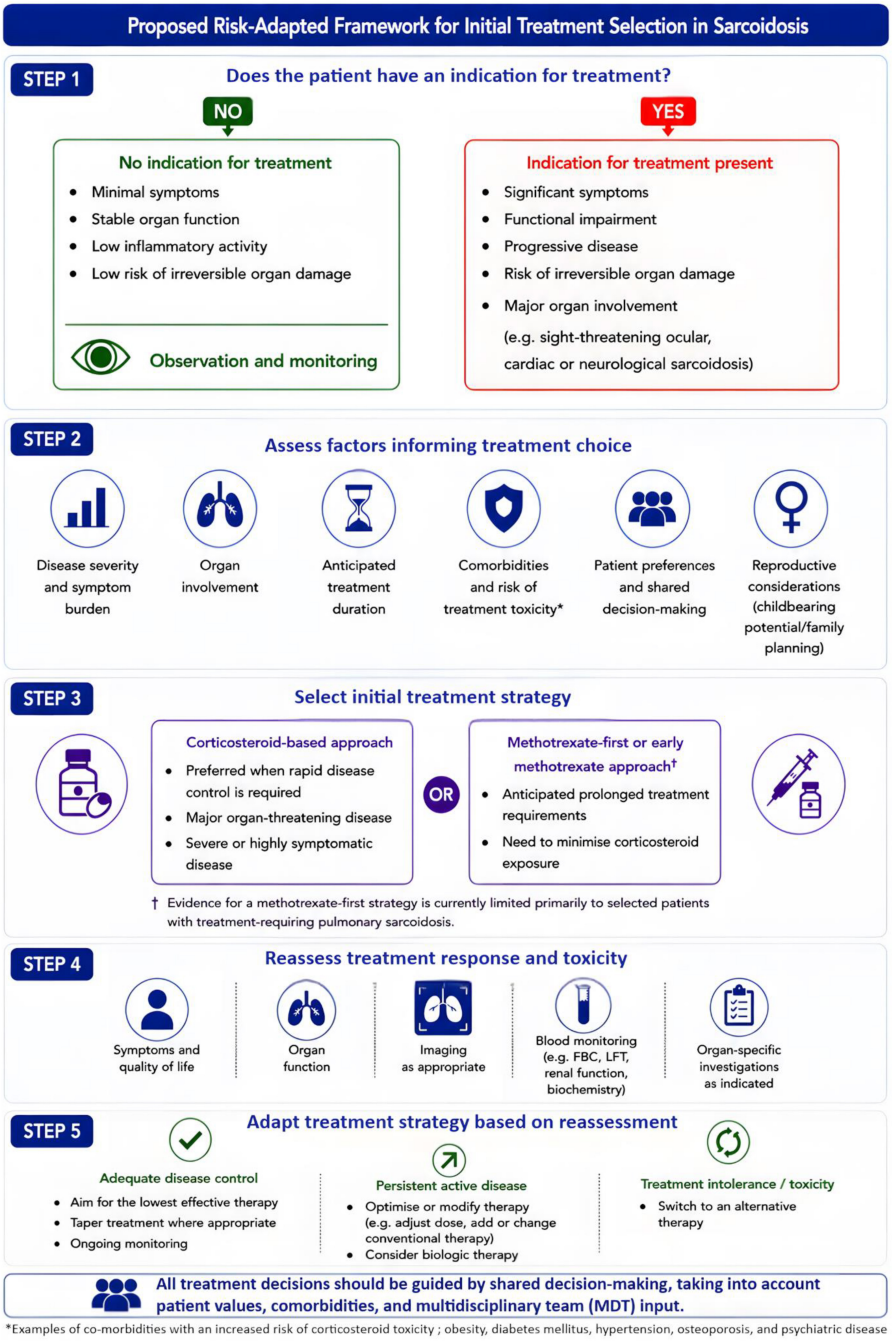
Proposed risk-adapted framework for initial treatment selection in sarcoidosis. Patients without an indication for treatment may be managed with observation and monitoring. For those requiring treatment, therapeutic decisions should incorporate disease severity, organ involvement, potential treatment duration, comorbidities, potential treatment-specific toxicities, reproductive considerations, and patient preferences. Reproductive considerations should be assessed before treatment initiation, as several therapeutic agents are contraindicated and appropriate pre-conception counseling is required. Initial therapy may comprise either a corticosteroid-based approach or, in selected patients, a methotrexate-first or early methotrexate strategy. Corticosteroids remain the standard first-line therapy in current guidelines and are preferred when rapid disease control is required or in major organ-threatening disease. Emerging evidence supports consideration of methotrexate as an alternative first-line option in selected patients with pulmonary sarcoidosis, particularly where prolonged treatment is anticipated or minimizing long-term corticosteroid exposure is a priority. Treatment response and toxicity should be reassessed regularly, with subsequent treatment modification, tapering where appropriate, or escalation of therapy according to disease activity and treatment tolerance. This framework reflects current evidence and guideline recommendations while recognizing the need for individualized treatment decisions. Figure created using BioRender.com. Some icons were generated using artificial intelligence based on prompts provided by the author.

Overall, the evidence supports corticosteroids for rapid disease control, methotrexate as the preferred steroid-sparing agent when prolonged therapy is anticipated, and infliximab as the best-supported biologic option for refractory or organ-threatening disease. A proposed risk-adapted framework integrating these principles is shown in Figure 2. Comparative data remain scarce for alternative conventional immunosuppressants and newer targeted therapies, leaving uncertainty regarding optimal treatment sequencing, duration of therapy, and patient selection beyond first-line treatment.

This review highlights the evolving treatment landscape in sarcoidosis and a growing recognition that management should move beyond the traditional corticosteroid-centered approach. Although corticosteroids remain a cornerstone of therapy because of their rapid anti-inflammatory effects and broad efficacy across organ systems, many patients require prolonged treatment and relapse after withdrawal is common. The contemporary challenge is to suppress disease activity whilst limiting cumulative treatment-related toxicity, achieved through earlier steroid-sparing strategies and more individualized decision-making.

The evidence supports a more nuanced appraisal of corticosteroid therapy; corticosteroids are effective for short-term disease control, particularly when rapid suppression of inflammation is required, but long-term use is frequently constrained by adverse effects. Furthermore, escalation to higher doses does not consistently translate into improved long-term outcomes across all patient groups. These observations have reinforced the importance of using the lowest effective dose, tapering treatment as soon as clinically feasible, and considering earlier introduction of steroid-sparing therapy when prolonged immunosuppression is anticipated.

Steroid-sparing immunosuppressive therapy now plays an increasingly central role in sarcoidosis management. Methotrexate remains the best-supported conventional agent and is widely regarded as the preferred second-line therapy. Emerging evidence also challenges traditional treatment sequencing, suggesting that methotrexate-based first-line strategies may provide comparable disease control while reducing cumulative corticosteroid exposure in selected patients. This raises important questions as to whether earlier or sole use at the outset of methotrexate should be considered in patients likely to require prolonged treatment, those at higher risk of corticosteroid toxicity, relapse or chronic progressive disease.

In contrast, there is limited evidence supporting alternatives such as azathioprine, mycophenolate mofetil, and leflunomide, as most knowledge is derived from observational studies, limiting confident comparisons between agents and reinforcing that treatment selection often depends on comorbidities, tolerability, clinician experience, and local practice.

Beyond conventional immunosuppression, the evidence base for biologic therapy is strongest for c inhibition, with infliximab representing the preferred biologic option for refractory disease or organ-threatening manifestations. However, the broader biologic and targeted therapy landscape remains relatively immature. Data supporting adalimumab are more limited, while evidence for non-TNF biologics and targeted synthetic therapies remains preliminary. Although early studies of newer agents, including JAK inhibitors and other emerging investigational therapies, suggest potential benefit in highly selected refractory cases, their role is currently constrained by small sample sizes, short follow-up periods, and a lack of comparative trials.

Interpretation of treatment studies is further complicated by the marked heterogeneity of sarcoidosis. Prognosis, relapse risk, and treatment responsiveness vary substantially according to clinical phenotype, organ involvement, inflammatory activity, and the extent of fibrosis. Clinical trials have also relied heavily on pulmonary function as primary endpoints. Although these measures are important, they may not adequately reflect extrapulmonary disease activity or patient-prioritized outcomes such as fatigue, functional limitation, and quality of life. This mismatch contributes to uncertainty when interpreting treatment benefit and may partly explain persistent variation in clinical practice despite broadly similar treatment principles.

Taken together, the available evidence supports an individualized, risk-adapted approach to treatment selection. Observation remains appropriate for many patients with mild disease and a low risk of progression. Corticosteroids remain essential when rapid disease control is required, particularly in the setting of major organ involvement. Earlier methotrexate introduction may be appropriate for patients likely to require prolonged therapy, those at increased risk of corticosteroid toxicity, or those with relapsing or chronic progressive disease. Biologic therapies are generally reserved for refractory disease and organ-threatening manifestations that fail to respond adequately to conventional immunosuppressive treatment. Treatment decisions should be guided not only by disease activity but also by anticipated treatment duration, comorbidities, patient preferences, and the long-term risks of therapy. This approach is broadly consistent with current guideline-recommended treatment pathways while highlighting important areas where evidence remains insufficient to guide optimal sequencing and selection of steroid-sparing and biologic therapies.

A key limitation of this review is that its conclusions are constrained by both the underlying literature and the review process itself. Included studies were heterogeneous with respect to phenotype definitions, organ involvement, and outcome measures; comparative head-to-head data were limited; and many studies were observational with variable risk of bias. Furthermore, differences in outcome selection, often emphasizing pulmonary physiology rather than multisystem or patient-centered outcomes, together with inconsistent follow-up durations, reduce the ability to draw firm conclusions regarding long-term effectiveness, relapse prevention, and the relative merits of competing steroid-sparing strategies.

## Emerging therapies and future directions

One of the major challenges in sarcoidosis management is the lack of reliable biomarkers capable of identifying patients at risk of aggressive disease, treatment response, or relapse. Although biomarkers such as ACE and soluble interleukin-2 receptor (sIL-2R) can be used in clinical practice, their performance is insufficient to support routine risk stratification or treatment selection (53).

Future research is therefore increasingly focused on integrated biomarker strategies that combine clinical phenotyping with molecular, immunological and imaging data. Multi-omic approaches with composite biomarker panels and imaging-integrated risk stratification models may improve identification of patients at risk of chronic progressive disease and facilitate a more personalized approach to treatment (126). Machine learning and artificial intelligence are increasingly being explored in sarcoidosis and related interstitial lung diseases to support disease phenotyping, prognostication and biomarker discovery, and personalized treatment selection in sarcoidosis; however, current applications remain early-stage and require external validation before routine clinical use (127, 128).

**FIGURE 3:**
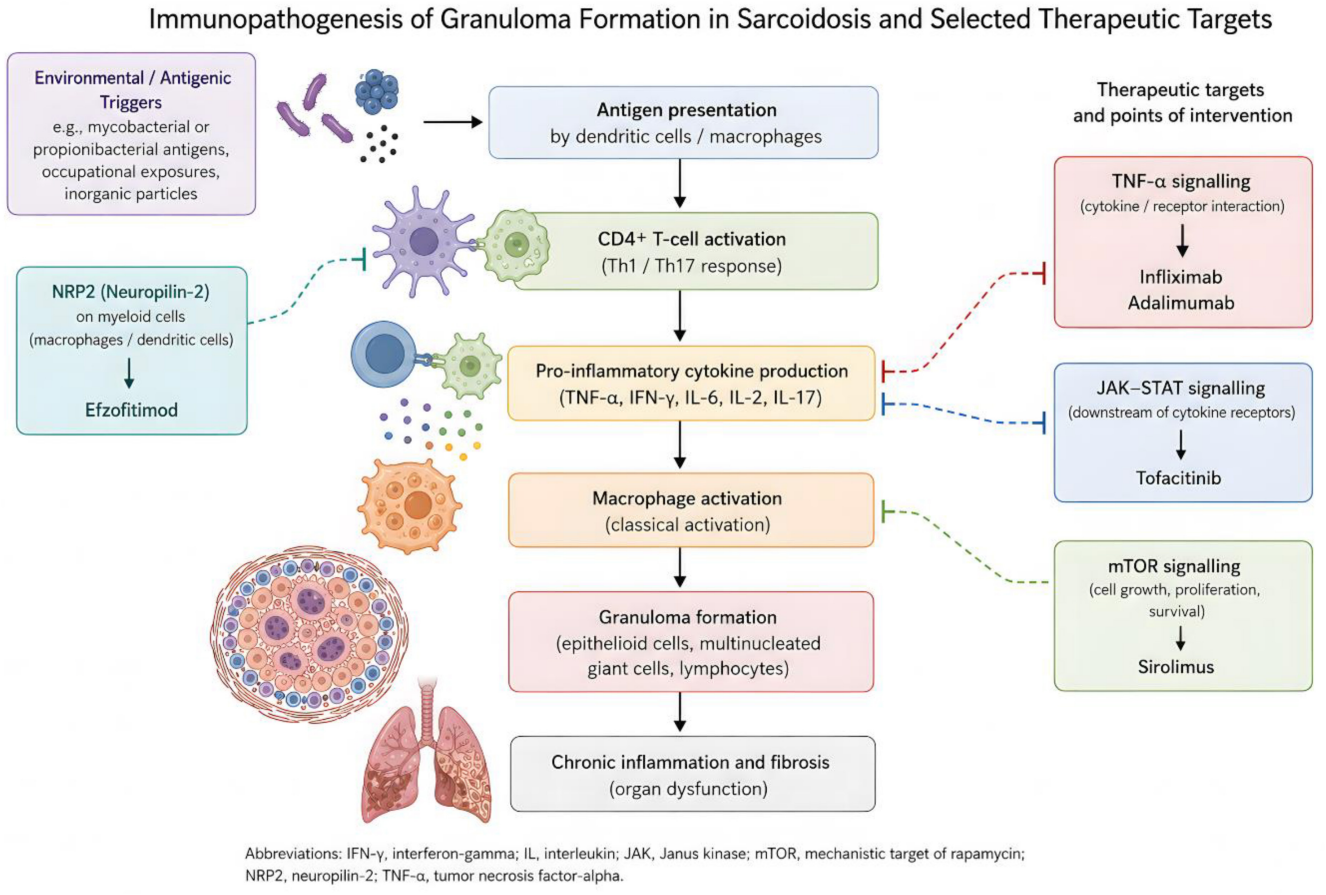
Immunopathogenesis of granuloma formation in sarcoidosis and selected therapeutic targets. Unknown environmental and/or antigenic triggers in genetically susceptible individuals activate antigen-presenting cells and CD4^+^ T lymphocytes, leading to cytokine production, macrophage activation, granuloma formation, and, in some patients, chronic inflammation, fibrosis, and organ dysfunction. The cytokines shown represent key inflammatory mediators in sarcoidosis pathogenesis, while the highlighted therapeutic targets correspond to selected therapies discussed in this review. The Figure is intended as a simplified schematic and does not include all pathogenic pathways or therapeutic targets. Figure created using artificial intelligence and modified by the authors using BioRender.com.

Advances in the understanding of sarcoidosis immunopathogenesis have identified several potential therapeutic targets beyond conventional immunosuppression (Figure 3). Janus kinase (JAK) inhibitors may have a role in reducing pro-inflammatory cytokines involved in granuloma formation through the Janus kinase–signal transducer and activator of transcription (JAK–STAT) signaling pathways (129). Clinical improvement with tofacitinib has been reported in patients with cutaneous and multisystem sarcoidosis; however, current evidence is limited to small studies, and further research is required to establish its long-term efficacy and safety (118, 130). Other intracellular pathways under investigation include mTOR signaling, which is thought to contribute to granuloma formation and may be a potential therapeutic target in sarcoidosis. Early clinical evidence suggests that systemic sirolimus may be effective in persistent glucocorticoid-refractory sarcoidosis (131, 132).

Beyond this, several novel immunomodulatory biologics are under investigation. Efzofitimod, a biologic targeting the neuropilin-2 receptor (NRP2), has shown potential clinical benefit in a small randomized controlled trial, including improvements in lung function and patient-reported outcomes (133); however, these findings remain exploratory and require confirmation in larger clinical trials. In contrast, several cytokine-targeted approaches have yielded mixed or negative results in sarcoidosis, including a lack of efficacy with IL-12/23 inhibition (ustekinumab), negative findings with IL-6 receptor blockade (sarilumab), and only limited benefit with IL-1 blockade (anakinra) despite improvements in inflammatory biomarkers (118).

Future progress will depend not only on expanding therapeutic options but also on improving treatment selection through more personalized approaches to care. Key priorities include the development and validation of predictive biomarkers, refined disease phenotyping, and biomarker-guided treatment algorithms. There is a particular need for adequately powered comparative effectiveness studies, including head-to-head trials evaluating first-line treatment strategies and studies defining the optimal sequencing and timing of corticosteroids, conventional immunosuppressants, biologics, and emerging targeted therapies. Future trials should also incorporate validated sarcoidosis-specific patient-reported outcome measures, given that traditional physiological endpoints may not fully capture symptom burden, fatigue, functional impairment, and quality of life. Collectively, these advances are essential to support a more personalized, risk-adapted approach to sarcoidosis management and improve long-term outcomes.

## Conclusion

Sarcoidosis remains a complex and heterogeneous disease, requiring treatment decisions that balance disease control against treatment-related toxicity. While corticosteroids remain the cornerstone of therapy, increasing recognition of their long-term adverse effects has driven growing interest in steroid-sparing and targeted treatment strategies. Contemporary management is increasingly moving toward personalized and risk-adapted approaches that consider disease phenotype, organ involvement, comorbidities, and patient preferences. Future progress will depend on improved risk stratification, validated biomarkers, high-quality comparative trials, and the development of targeted therapies that enable more precise treatment selection and improved long-term outcomes.
